# SCD1 Expression Is Dispensable for Hepatocarcinogenesis Induced by AKT and Ras Oncogenes in Mice

**DOI:** 10.1371/journal.pone.0075104

**Published:** 2013-09-19

**Authors:** Lei Li, Chunmei Wang, Diego F. Calvisi, Matthias Evert, Maria G. Pilo, Lijie Jiang, Mariia Yuneva, Xin Chen

**Affiliations:** 1 Department of Bioengineering and Therapeutic Sciences, University of California San Francisco, San Francisco, California, United States of America; 2 School of Pharmacy, Tongji Medical College, Huazhong University of Science and Technology, Wuhan, Hubei, China; 3 Department of Clinical and Experimental Medicine, University of Sassari, Sassari, Italy; 4 Institute of Pathology, University of Greifswald, Greifswald, Germany; 5 G.W. Hooper Research Foundation, University of California San Francisco, San Francisco, California, United States of America; 6 Liver Center, University of California San Francisco, San Francisco, California, United States of America; University of Texas MD Anderson Cancer Center, United States of America

## Abstract

Increased *de novo* lipogenesis is one of the major metabolic events in cancer. In human hepatocellular carcinoma (HCC), *de novo* lipogenesis has been found to be increased and associated with the activation of AKT/mTOR signaling. In mice, overexpression of an activated form of AKT results in increased lipogenesis and hepatic steatosis, ultimately leading to liver tumor development. Hepatocarcinogenesis is dramatically accelerated when AKT is co-expressed with an oncogenic form of N-Ras. SCD1, the major isoform of stearoyl-CoA desaturases, catalyzing the conversion of saturated fatty acids (SFA) into monounsaturated fatty acids (MUFA), is a key enzyme involved in *de novo* lipogenesis. While many studies demonstrated the requirement of SCD1 for tumor cell growth *in vitro*, whether SCD1 is necessary for tumor development *in vivo* has not been previously investigated. Here, we show that genetic ablation of *SCD1* neither inhibits lipogenesis and hepatic steatosis in AKT-overexpressing mice nor affects liver tumor development in mice co-expressing AKT and Ras oncogenes. Molecular analysis showed that SCD2 was strongly upregulated in liver tumors from AKT/Ras injected *SCD1* ^*-/-*^ mice. Noticeably, concomitant silencing of SCD1 and SCD2 genes was highly detrimental for the growth of AKT/Ras cells *in vitro*. Altogether, our study provides the evidence, for the first time, that SCD1 expression is dispensable for AKT/mTOR-dependent hepatic steatosis and AKT/Ras-induced hepatocarcinogenesis in mice. Complete inhibition of stearoyl-CoA desaturase activity may be required to efficiently suppress liver tumor development.

## Introduction

Hepatocellular carcinoma (HCC) is the fifth most common cancer worldwide [1]. HCC is generally a fatal disease. Treatment options for HCC are limited and generally ineffective [2-4]. Therefore, the investigation of the molecular pathogenesis of HCC is necessary for the development of new targeted therapies against this deadly disease.

Accumulating evidence indicates that metabolic imbalance is one of the hallmarks of cancer [5,6]. In particular, aberrant lipid metabolism in the form of increased *de novo* fatty acid synthesis is an important feature of malignant transformation and tumor progression [7,8]. Indeed, rapidly-proliferating cancer cells often display a robust program of fatty acid synthesis that is necessary to fuel membrane production and lipid-based post-translational modifications [7,8].

One key regulator of the fatty acid composition of cellular lipids is Stearoyl-CoA desaturase (SCD), also known as fatty acyl-CoA delta-9 desaturase. SCD catalyzes the introduction of the first double bond in the cis-delta-9 position of several saturated fatty (SFA) acyl-CoAs, principally palmitoyl-CoA and stearoyl-CoA, to yield monounsaturated fatty acid (MUFA), palmitoleic acid (16:1), and oleic acid (18:1), respectively [9]. In the mouse, four SCD isoforms (SCD1-SCD4) have been identified, whereas in humans only two genes (SCD1 and SCD5) have been isolated, with human SCD1 being co-orthologous to the four murine genes [10]. Recent studies suggest that SCD1 plays critical role(s) along malignant transformation and tumor cell growth both in humans and rodents [11]. For instance, SCD1 upregulation has been detected in breast, prostate, colon, and esophageal cancers [12], with elevated levels of SCD1 being associated to poor prognosis in breast cancer patients [13]. In addition, silencing of SCD1 expression restrained the growth and promoted apoptosis of prostate and colon cancer cells [14]. Furthermore, depletion of SCD1 inhibited oncogene induced malignant transformation of human primary fibroblasts [15]. However, virtually all the functional studies on SCD1 were performed *in vitro* using tumor cell lines. Thus, it remains unknown whether SCD1 expression is required for tumor development and progression *in vivo*.

The v-akt murine thymoma viral oncogene homolog (AKT)/mammalian target of Rapamycin (mTOR) pathway is a central regulator of multiple cellular processes, including metabolism, growth, proliferation, and survival. Recent studies identified the AKT/mTOR signaling cascade as the central regulator of lipid homeostasis, including *de novo* lipogenesis [15]. Specifically, sterol regulatory element-binding proteins (SREBPs), the major transcriptional factors in regulating fatty acid synthesis, are pivotal effectors downstream of mTOR complex 1 (mTORC1) [16,17,18]. In the liver, it has been previously shown that overexpression of an activated form of AKT leads to increased *de novo* lipogenesis and hepatic steatosis via the mTORC1/SREBP1 pathway [19]. Rapid liver tumor formation is observed when AKT is co-expressed with the oncogenic form of N-Ras in mice, which will be referred to as AKT/Ras tumor model in this study [20]. Of note, preneoplastic and neoplastic liver cells from AKT/Ras mice display elevated *de novo* lipogenesis, associated with intracellular lipid accumulation and strong activation lipogenic pathway genes, including SCD1 [20].

In the present investigation, we assessed the functional contribution of SCD1 *in vivo* both on hepatic steatosis driven by AKT/mTOR and liver cancer development induced by AKT/Ras co-expression. Our results indicate that SCD1 is not essential for AKT/mTOR-dependent hepatic steatosis and AKT/Ras-induced hepatocarcinogenesis in mice.

## Materials and Methods

### Ethics Statement

Mice were housed, fed, and monitored in accordance with protocols approved by the committee for animal research at the University of California, San Francisco (IACUC approval number: AN087765). Mice were monitored closely for liver tumor development. Mice with noticeable swelling abdominal mass or with a body condition score 2 or less were euthanized by carbon dioxide inhalation followed by cervical dislocation according to the approved IACUC protocol.

### Constructs

All the constructs, including pT3-Caggs-RasV12, pT3-EF1a-myr-AKT and pCMV/sleeping beauty transposase (SB) used for mouse injection were previously described [20,21]. Plasmids were purified using the Endotoxin-free Maxiprep kit (Sigma, St. Louis, MO).

### Hydrodynamic injection and mouse monitoring

*SCD1* ^*+/-*^ mice [22] were purchased from the Jackson Laboratory (Stock number: 006201). *SCD1* ^*+/-*^ mice were back-crossed with wild-type FVB/N mice for at least five generations. After back-crossing, the *SCD1* ^*+/-*^ mice were then inter-crossed to obtain *SCD1* ^*-/-*^ mice as well as control wild-type littermates. Genotyping was performed by polymerase chain reaction (PCR) on genomic DNA from tail clips as described by the Jackson Laboratory. Hydrodynamic gene delivery was performed as described previously [23,24]. Briefly, 10µg of the plasmids encoding myr-AKT and/or RasV12 gene along with sleeping beauty transposase in a ratio of 25:1 were diluted in 2 ml saline (0.9% NaCl) for each mouse. Saline solution was filtered through a 0.22 µm filter and injected into the lateral tail vein of 6 to 8-week-old mice in 5–7 seconds.

### Immunohistochemical staining

Liver tissues were fixed in 4% paraformaldehyde overnight at 4°C and embedded in paraffin. Antigen retrieval was performed in 10 mM sodium citrate buffer (pH 6.0) by placement in a microwave on high for 10 min, followed by a 20-min cool down at room temperature. After a blocking step with the 5% goat serum and Avidin-Biotin blocking kit (Vector Laboratories, Burlingame, CA), the slides were incubated with primary antibodies overnight at 4°C. Slides were then subjected to 3% hydrogen peroxide for 10 min to quench endogenous peroxidase activity and subsequently the biotin conjugated secondary antibody was applied at a 1:400 dilution for 30 min at room temperature. Anti-SCD1, FASN, HA, p-AKT, p-ERK, p-mTOR and p-RPS6 antibodies were all obtained from Cell Signaling Technology Inc. Detection was performed with the ABC-Elite peroxidase kit (Vector Laboratories) by using the DAB substrate kit (Dakocytomation).

### Oil-Red-O staining

The Oil Red O staining was performed with the Oil Red O stain kit (American MasterTech), according to the manufacturer’s protocol. ImageJ software was used for quantification of Oil Red O positive area as percentage of threshold area.

### Western blotting

Liver tissues and HCC cells were processed as previously reported [22]. Nitrocellulose membranes were probed with specific primary antibodies followed by horseradish peroxidase secondary antibodies (Jackson ImmunoResearch Laboratories Inc., West Grove, PA). β-actin was used as a loading control and proteins were revealed with the Super Signal West Pico (Pierce Chemical Co., New York, NY). Anti-SCD1, FASN, AKT, p-AKT, ERK1/2, p-ERK1/2, RPS6, and p-RPS6 antibodies were purchased from Cell Signaling Technology; anti-SCD2 and β-actin antibodies were purchased from Santa Cruz Biotechnology.

### Quantitative reverse transcription-polymerase chain reaction (qRT-PCR)

Primers for mouse *SCD1*, *SCD2*, *SCD3*, *SCD4*, and *RNR-18* genes were purchased from Applied Biosystems (Foster City, CA). PCR reactions were performed with 100 ng of cDNA from each mouse sample and the AKT/Ras cell line, using an ABI Prism 7000 Sequence Detection System and TaqMan Universal PCR Master Mix (Applied Biosystems). Cycling conditions were: 10 min of denaturation at 95°C and 40 cycles at 95°C for 15 s and at 60°C for 1 min. Quantitative values were calculated by using the PE Biosystems Analysis software and expressed as N target (NT). NT = 2^-ΔCt^, wherein ΔCt value of each sample was calculated by subtracting the average Ct value of the target gene from the average Ct value of the *RNR-18* gene.

### Lipid analysis

Lipid analysis was performed by the MMPC core at Vanderbilt University. In brief, lipids were extracted using the method of Folch-Lees [25]. Triglycerides and cholesteryl esters were scraped from the plates and methylated using BF3/methanol as described by Morrison and Smith [26]. Methylated fatty acids were extracted and analyzed by gas chromatography. Gas chromatographic analyses were carried out on an Agilent 7890A gas chromatograph equipped with flame ionization detectors, a capillary column (SP2380, 0.25 mm x 30 m, 0.25 µm film; Supelco, Bellefonte, PA). Helium was used as a carrier gas. The oven temperature was programmed from 160 °C to 230 °C at 4 °C/min. Fatty acid methyl esters were identified by comparing the retention times to those of known standards. Inclusion of lipid standards with odd chain fatty acids permitted quantitation of the amount of lipid in the sample. Dipentadecanoyl phosphatidylcholine (C15: 0), diheptadecanoin (C17: 0), trieicosenoin (C20: 1), and cholesteryl eicosenoate (C20: 1) were used as standards.

### Cell line and Treatments

The primary AKT/Ras HCC cell line, isolated from a HCC developed in an AKT/Ras mouse, was previously described [19]. The cell line was maintained as monolayer culture in Dulbecco’s modified Eagle medium supplemented with 10% fetal bovine serum. 2.0 x 10^3^cells were plated per well in 96-well plate and grown for 12 h. For silencing experiments, the AKT/Ras cell line was then serum deprived for 24 h and treated with small interfering RNA (siRNA) against SCD1 and/or SCD2 genes (Santa Cruz Biotechnology) according to the manufacturer’s recommendations, and incubated for 24 and 48 hours. To assess cell proliferation, AKT/Ras cells were plated at the concentration of 2.0 x 10^3^/well in 96-well plates, allowed to attach and adjust for the next 12 hours (which corresponds to the 0 hour time point in our graphs), and grown for the additional 24 and 48 hours. The proliferation was assessed at these three time points (0, 24, and 48 hours) with the BrdU Cell Proliferation Assay Kit (Cell Signaling Technology) by measuring the absorbance at 450 nm following the manufacturer’s protocol. To measure apoptosis, HCC cell lines were plated at the concentration of 2.0 x 10^3^ /well in 96-well plates, incubated for 12 hours, and then subjected to 24-hour serum deprivation (which corresponded to the 0 hour time point in the apoptosis graphs). Apoptosis was assessed at three time points (0, 24, and 48 hours) with the Cell Death Detection Elisa Plus Kit (Roche Molecular Biochemicals, Indianapolis, IN) by measuring the absorbance at 405 nm, following the manufacturer’s instructions.

### Statistical analysis

Student’s t and Tukey–Kramer tests were used to evaluate statistical significance. Values of P <0.05 were considered significant. Data are expressed as mean ± SD.

## Results

### Ablation of *SCD1* does not inhibit AKT induced hepatic steatosis

**Figure 1:**
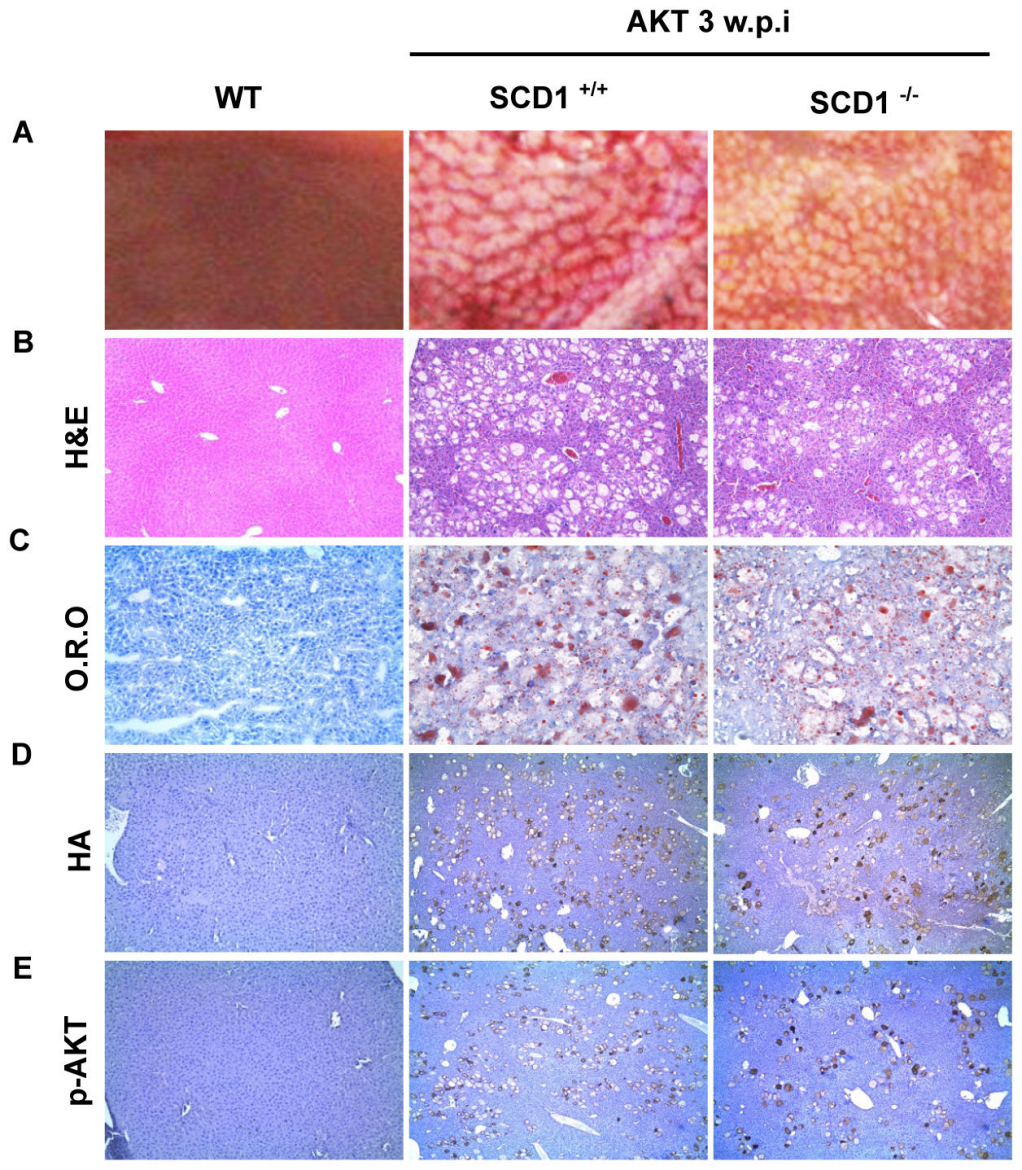
myr-AKT induces hepatic steatosis in both *SCD1* ^*+/+*^ and *SCD1* ^*-/-*^ mice 3 weeks post injection. (A) Gross images, (B) H&E staining, (C) Oil Red O staining, (D) and (E) Immunostaining of HA (D) and p-AKT (E) staining of liver tissues from uninjected wild-type mice (WT), AKT injected *SCD1* ^*+/+*^ and AKT injected *SCD1* ^*-/-*^ mice. w.p.i: weeks post injection. Original magnification in B, C, D and E is 100x.

Previously, we found that overexpression in the mouse liver of a myristoylated/activated form of AKT1 (myr-AKT1 with C-terminal HA tag), which will be here referred to as AKT, leads to extensive hepatic steatosis and increased expression of enzymes of the lipogenic pathway, such as FASN, ACAC, ACLY, and SCD1 [19]. To investigate the role of SCD1 in AKT induced hepatic steatosis, we generated *SCD1* KO mice by breeding *SCD1* ^*+/-*^ mice in the FVB/N background. *SCD1* ^*-/-*^ mice were born as expected ratio. They exhibited cutaneous abnormalities and narrow eye fissures, consistent with the phenotype previously described for these mice [22]. Next, we hydrodynamically injected AKT into *SCD1* ^*-/-*^ and *SCD1* ^*+/+*^ mice. Mice were euthanized 3 weeks post injection. Grossly, liver appeared to be pale and spotty in both *SCD1* ^*-/-*^ and *SCD1* ^*+/+*^ mice (Figure 1A). Histological analysis revealed extensive steatosis with balloon-like lipid-storing hepatocytes, scattered throughout the liver regardless of SCD1 status (Figure 1B). Oil-Red-O staining further demonstrated extensive lipid droplet formation in the liver of *SCD1* ^*-/-*^ and *SCD1* ^*+/+*^ mice (Figure 1C). Quantification of Oil-Red-O staining demonstrated no significant difference in lipid content between the two mouse strains (% Threshold Area: 13.88±2.43% in *SCD1* ^*+/+*^ mice; and 13.45±2.05% *SCD1* ^*-/-*^ mice). The expression of ectopically injected AKT was successfully identified in the altered hepatocytes via HA or p-AKT immunostaining (Figure 1D and E).

Altogether, our results suggest that SCD1 expression is not essential for AKT induced hepatic steatosis.

### AKT/Ras induces liver cancer formation in *SCD1* null mice

**Figure 2:**
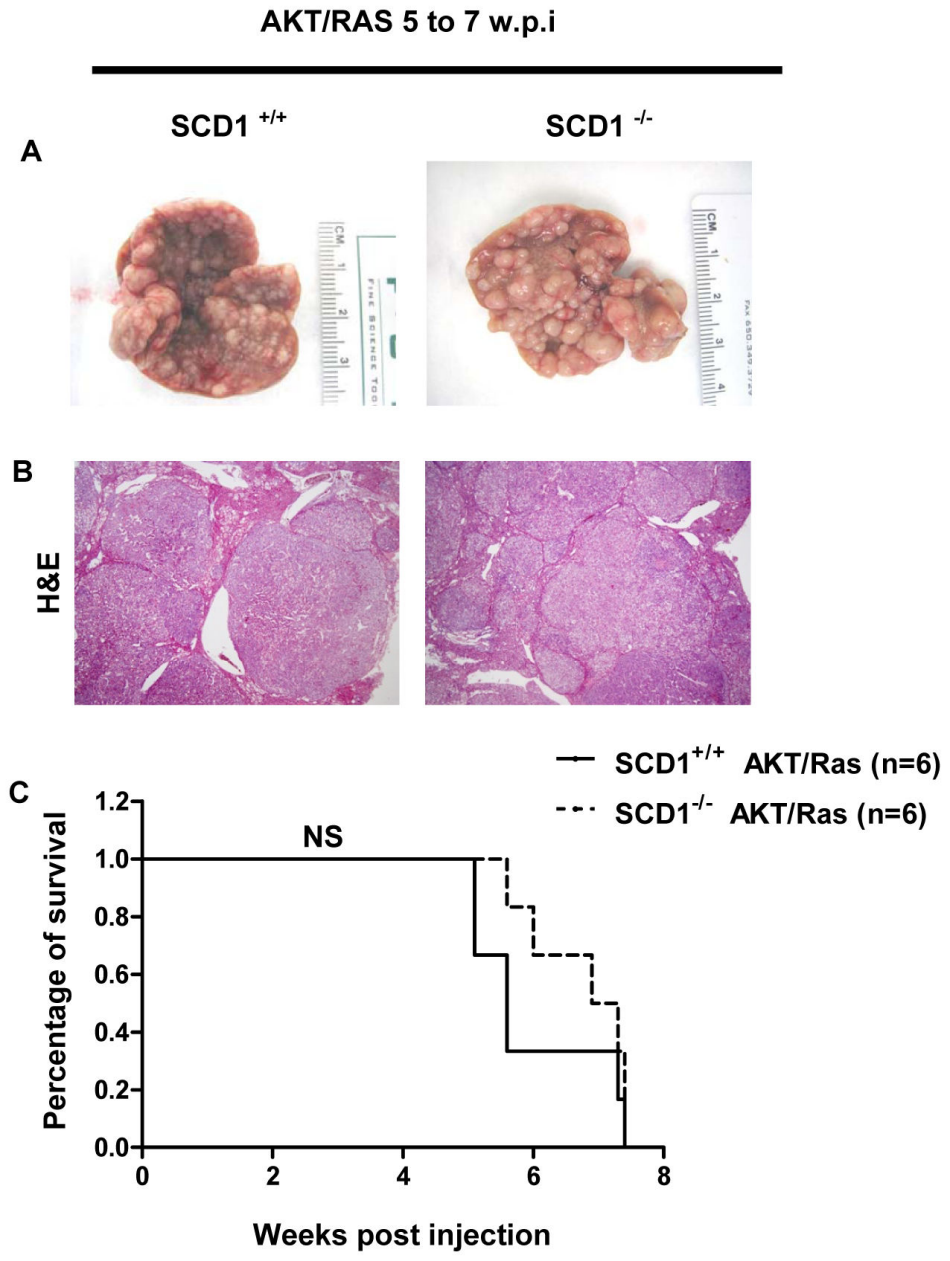
AKT/Ras induces liver cancer formation in both *SCD1* ^*+/+*^ and *SCD1* ^*-/-*^ mice. (A) Gross images, (B) H&E staining, and (C) Survival Curve of AKT/Ras injected *SCD1* ^*+/+*^ and *SCD1* ^*-/-*^ mice. w.p.i: weeks post injection. NS: not significant. Original magnification in B is 20x.

**Figure 3:**
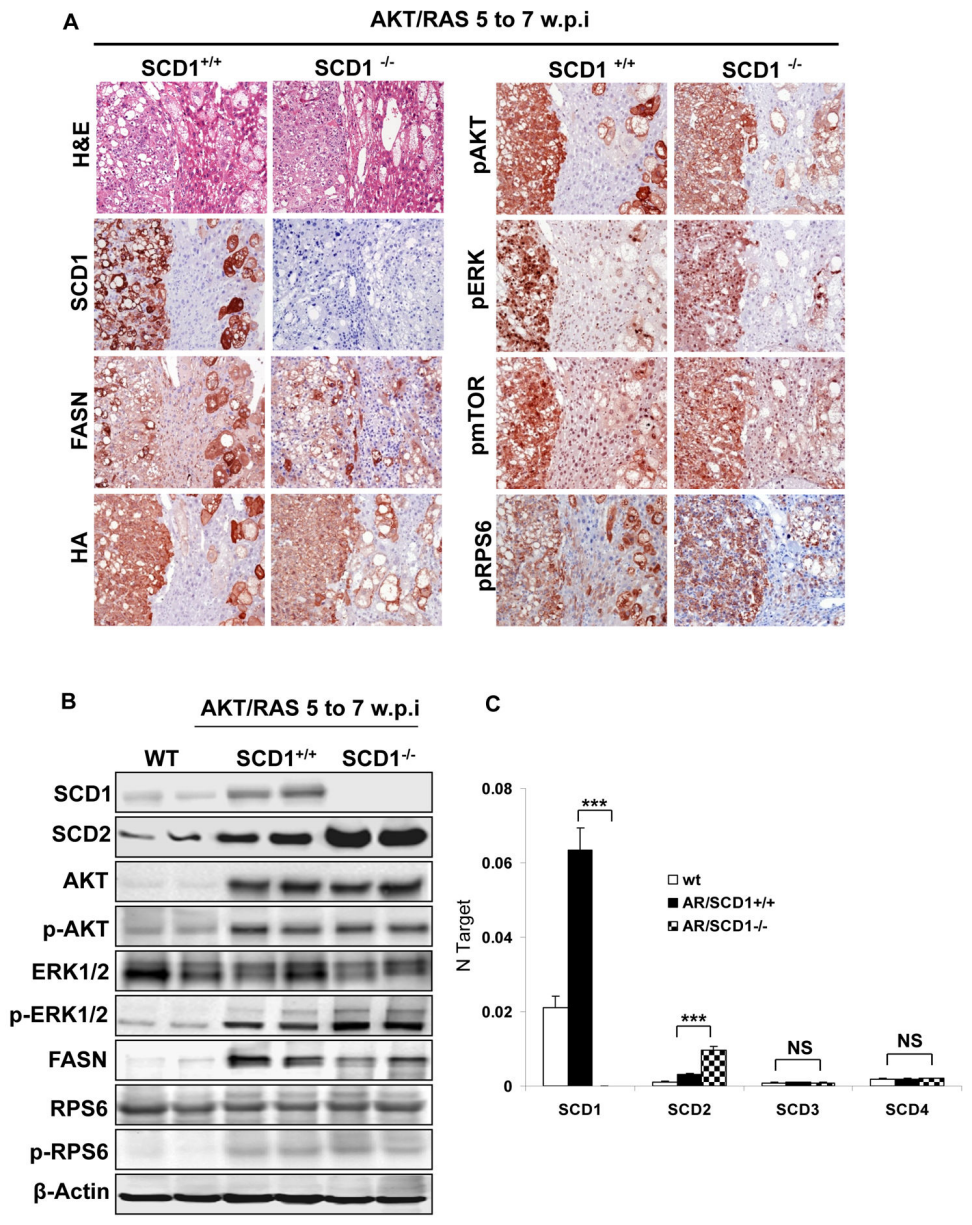
Molecular characterization of AKT/Ras induces liver cancer formation in *SCD1* ^*+/+*^ and *SCD1* ^*-/-*^ mice. (A) H&E and immunostaining of AKT/Ras tumors from *SCD1* ^*+/+*^ and *SCD1* ^*-/-*^ mice. In the immunostainings, serial sections of a hepatocellular tumor (left part) and scattered preneoplastic cells (right part) are shown. (B) Western blotting analysis of AKT/Ras tumors from *SCD1* ^*+/+*^ and *SCD1* ^*-/-*^ mice. (C) Quantification of the levels of SCD2-SCD4 genes in AKT/Ras tumor tissues by real-time qRT-PCR analysis. w.p.i: weeks post injection. Original magnification in A is 100x. *** P<0.001. NS: not significant.

Next, we investigated whether SCD1 is required for AKT/Ras induced liver cancer development. For this purpose, we co-injected myr-AKT1 and NRasV12 into *SCD1* ^*-/-*^ and *SCD1* ^*+/+*^ mice. We found that forced overexpression of AKT and Ras oncogenes synergized to promote liver tumor formation in both *SCD1* ^*+/+*^ and *SCD1* ^*-/-*^ mice (Table 1 and Figure 2A and B). Specifically, large tumors developed in both strains of mice and mice needed to be euthanized between 5 to 7 weeks post-injection (Table 1 and Figure 2C). Histological evaluation of the liver tissue showed that preneoplastic lesions and tumors occupied almost completely the liver parenchyma (Figure 2B). In accordance with our previous study [22], numerous frankly malignant tumors, mostly pure HCC and, to a lesser degree, mixed hepatocellular/cholangiocellular or pure cholangiocellular carcinomas developed (Figure 2B) in both *SCD1* ^*-/-*^ and *SCD1* ^*+/+*^ mice. No statistical differences between *SCD1* ^*-/-*^ and *SCD1* ^*+/+*^ mice were detected as concerns liver weight (Table 1), proliferation (25.8 ± 4.6 *vs.* 23.6 ± 5.6, respectively; P=0.2), and apoptosis (2.4 ± 1.3 *vs.* 2.1 ± 1.8, respectively; P=0.1). Loss of SCD1 protein expression was confirmed in AKT/Ras tumors from *SCD1* ^*-/-*^ mice (Figure 3). At the molecular level, tumors from *SCD1* ^*-/-*^ and *SCD1* ^*+/+*^ showed an equivalent activation of the AKT/mTOR (p-AKT, p-mTOR and p-RSP6) and Ras/MAPK (p-ERK) pathways, as assessed by immunohistochemistry (Figure 3A). However, a slight increase of activated/phosphorylated ERK proteins was detected by western blotting (Figure 3B).

**Table 1:** Ablation of *SCD1* does not inhibit AKT/Ras induced liver cancer development.

**Code**	**Genotype**	**Gender**	W.P.I (*)	**liver weight**	**body weight**	**Ratio (#**)
WT AKTRas 1	*SCD1* ^*+/+*^	Female	7.3	8.5	24.2	0.35
WT AKTRas 2	*SCD1* ^*+/+*^	Male	7.4	12	31	0.39
WT AKTRas 3	*SCD1* ^*+/+*^	Female	5.6	11.2	36.3	0.31
WT AKTRas 4	*SCD1* ^*+/+*^	Female	5.6	8.8	34.2	0.26
WT AKTRas 5	*SCD1* ^*+/+*^	Male	5.1	15.4	35.7	0.43
WT AKTRas 6	*SCD1* ^*+/+*^	Male	5.1	13.6	35	0.39
KO AKTRas 1	*SCD1* ^*-/-*^	Female	6.9	8	31.4	0.25
KO AKTRas 2	*SCD1* ^*-/-*^	Female	7.3	9.8	26.1	0.38
KO AKTRas 3	*SCD1* ^*-/-*^	Male	7.4	10	31.9	0.31
KO AKTRas 4	*SCD1* ^*-/-*^	Male	7.4	6.2	35.8	0.17
KO AKTRas 5	*SCD1* ^*-/-*^	Female	5.6	10.1	30	0.34
KO AKTRas 6	*SCD1* ^*-/-*^	Female	6	7.3	32.3	0.23

* refers to weeks post injection; # refers to the ratio of liver weight to body weight.

In summary, the present data demonstrate that SCD1 expression is dispensable for AKT/Ras induced hepatocarcinogenesis.

### Increased expression of SCD2 in AKT/Ras induced liver tumor tissues

In the mouse, 3 additional SCD isoforms (SCD2-SCD4) have been identified [10]. Among them, SCD2 is expressed in the normal liver [27] and is responsible for the stearoyl-coeznyme A desaturase activity both in the developing mouse liver [28] and in the *SCD1* ^*-/-*^ mouse liver [28], whereas the role of SCD3 and SCD4 remains unknown [10]. Thus, we examined the levels of SCD2-SCD4 genes in AKT/Ras tumor tissues by real-time RT-PCR analysis (Figure 3C). We found that SCD2 expression was increased in AKT/Ras liver tumors from *SCD1* ^*+/+*^ mice when compared with normal livers from wild-type mice. A further, strong upregulation of SCD2 mRNA was detected in AKT/Ras tumors from *SCD1* ^*-/-*^ mice (Figure 3C). In contrast, levels of SCD3 and SCD4 were equivalent in normal livers and AKT/Ras liver tumors developed in *SCD1* ^*+/+*^ and *SCD1* ^*-/-*^ mice (Figure 3C). The pattern of SCD2 expression was confirmed at protein level by western blotting (Figure 3B). These results suggest that high expression of SCD2 might compensate for the loss of SCD1 along AKT/Ras driven hepatocarcinogenesis.

### Distinct lipid profiles in AKT/Ras liver tumors from *SCD1* ^+/+^ and *SCD1* ^-/-^ mice

**Figure 4:**
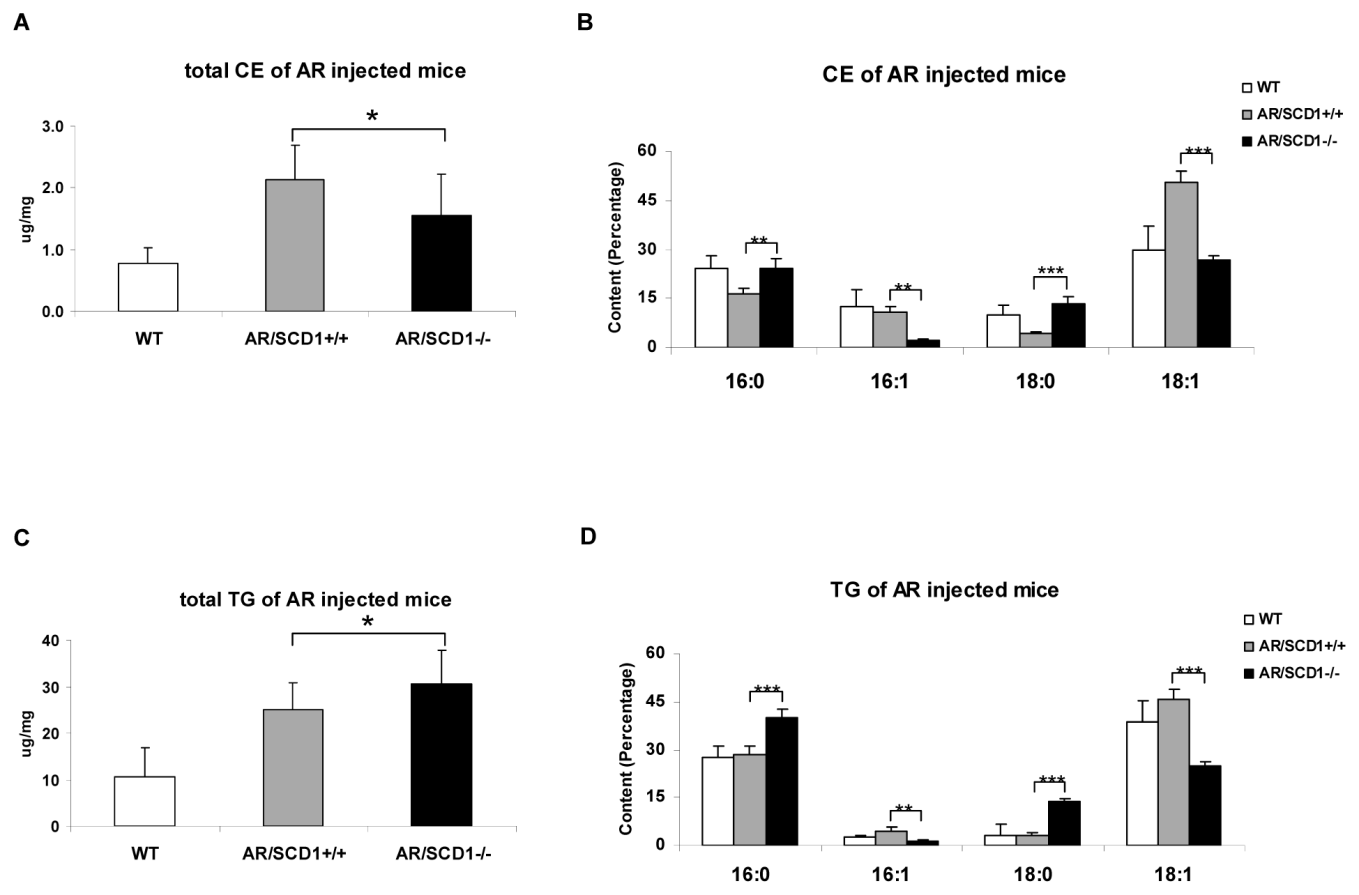
Lipid profiles of normal liver and liver tumors from AKT/Ras induced liver tumors in *SCD1* ^*+/+*^ and *SCD1* ^*-/-*^ mice. (A) Total Cholesterol ester (CE), (B) major fatty acids composition of Cholesterol ester, (C) Total Triglycerides (TG), and (D) major fatty acids composition of Triglycerides. At least 4 animals in each group were assayed. Student’s t test: *P<0.05; **P<0.01; ***P<0.001.

Next, we sought to determine whether the upregulation of SCD2 completely compensated for the loss of *SCD1* in terms of stearoyl-CoA desaturase activity in AKT/Ras tumor tissues. For this purpose, we investigated the fatty acid composition of AKT/Ras tumors in *SCD1* ^*+/+*^ and *SCD1* ^*-/-*^ mice. We found that while both triglycerides (TG) and cholesteryl esters (CE) levels were elevated in AKT/Ras tumors developed in both *SCD1* ^*+/+*^ and *SCD1* ^*-/-*^ mice (Figure 4A and C), the composition of the fatty acids in the TG and CE pools were quite distinct. Specifically, we found that the percentage of MUFA was significantly lower, whereas SFA was significant higher in AKT/Ras tumors from *SCD1* ^*-/-*^ mice (Figure 4B and D). This resulted in a significant altered MUFA versus SFA ratio in AKT/Ras liver tumors from *SCD1* ^*+/+*^ and *SCD1* ^*-/-*^ mice (Table 2). For instance, the average ratio of 18:1 to 18: 0 in the CE pool was 3.92 in the wild-type liver and increased to 11.59 in AKT/Ras tumor CE pool, consistent with the increased stearoyl-CoA desaturase activity in the tumor tissues. However, the average ratio dropped to 2.08 in AKT/Ras tumor from *SCD1* ^*-/-*^ mice.

**Table 2:** Major Fatty acid composition ratio of wildtype liver tissues as well as AKT/Ras induced liver tumors in *SCD1* ^*+/+*^ and *SCD1* ^*-/-*^ mice.

**CE**	**16:1/16:0**	**18:1/18:0**	**TG**	**16:1/16:0**	**18:1/18:0**
**WT**	0.51 ± 0.24	3.92 ± 1.60	**WT**	0.11 ± 0.02	12.84 ± 1.61
***AR/SCD1*^*+/+*^**	0.65 ± 0.09	11.59 ± 1.47	***AR/SCD1*^*+/+*^**	0.15 ± 0.04	15.95 ± 5.90
***AR/SCD1*^*-/-*^**	0.09 ± 0.01	2.08 ± 0.29	***AR/SCD1*^*-/-*^**	0.03 ± 0.01	1.84 ± 0.17

Values are means ± SD, n =6. Only the major fatty acids composition ratio were presented (P<0.05)

CE: Cholesterol Ester; TG: Triglyceride

WT: Normal liver from wild-type miceAR/SCD1^+/+^: AKT/Ras liver tumors from SCD1^+/+^ mice*AR/SCD1* ^- ^/ : AKT/Ras liver tumors from *SCD1* ^*-/-*^ mice

Taken together, our findings indicate that the upregulation of SCD2 in AKT/Ras tumor tissues cannot completely compensate for the loss of SCD1 expression in terms of liver lipid profile. Nevertheless, the aberrant fatty acid composition in the TG and CE pools consequent to SCD1 depletion does not significantly affect AKT/Ras induced hepatocarcinogenesis.

### Concomitant silencing of SCD1 and SCD2 strongly inhibits AKT/Ras cell growth *in vitro*

**Figure 5:**
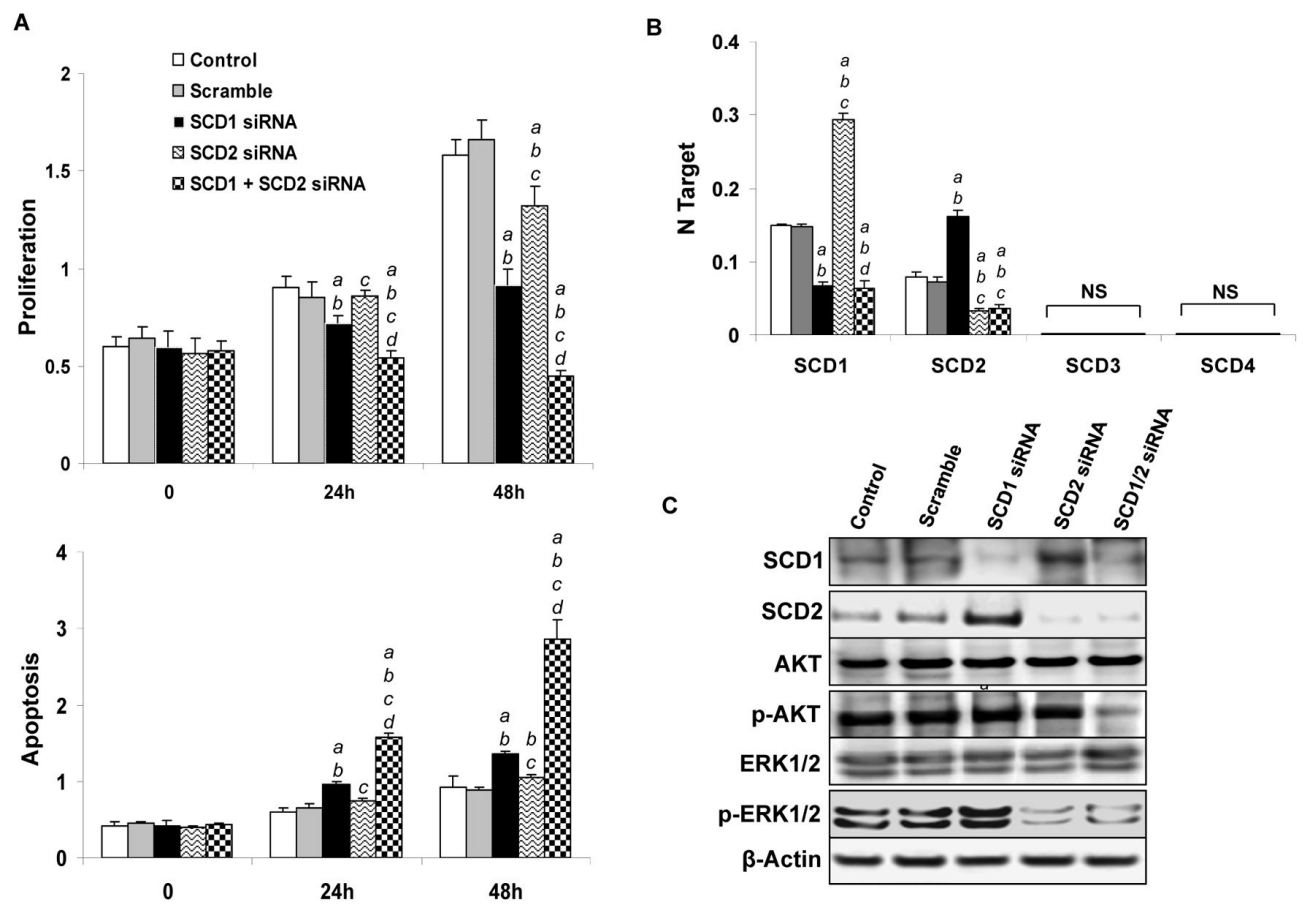
Concomitant silencing of SCD1 and SCD2 strongly inhibits AKT/Ras-induced cell growth *in vitro*. (A) Effect of silencing of SCD1 and SCD2, either alone or in combination, in AKT/Ras-induced proliferation and apoptosis. (B) qRT-PCR analysis of the levels of SCD2-SCD4 genes in AKT/Ras cells transfected with scramble siRNA, SCD1 siRNA, SCD2 siRNA or SCD1 plus SCD2 siRNA. (C) Western blot analysis of SCD1 and SCD2 protein expression AKT/Ras cells transfected with scramble siRNA, SCD1 siRNA, SCD2 siRNA or SCD1 plus SCD2 siRNA. Tukey-Kramer test: P < 0.005 *a*, vs. control (untreated cells); *b*, vs. scramble siRNA; *c*, vs. SCD1 siRNA; *d*, vs. SCD2 siRNA. Results 24 hours after silencing are shown. Results 48 hours after silencing not shown have no difference with 24 hours. Experiments *in* *vitro* were conducted at least three times in triplicate.

To gain further insights into the role of SCD2 in AKT/Ras driven growth, the AKT/Ras cell line was subjected to silencing of SCD1 and/or SCD2 genes (Figure 5) by using specific siRNAs. Silencing of either SCD1 or SCD2 gene led to a decrease in proliferation and increase of apoptosis (Figure 5A). At the molecular level, silencing of SCD1 resulted in a remarkable upregulation of SCD2 mRNA (Figure 5B) and protein (Figure 5C) in AKT/Ras cells, further supporting the existence of a compensatory mechanism triggered by SCD1 loss. No changes in SCD3 and SCD4 mRNA levels were detected following SCD1 silencing (Figure 5B). Of note, induction of SCD2 was accompanied by a slight upregulation of phosphorylated/activated ERK proteins, whereas levels of AKT activation were not affected by SCD1 silencing (Figure 5C). A slight upregulation of SCD1, but not SCD3 and SCD4 mRNA (Figure 5B), and SCD1 protein (Figure 5C) was observed in AKT/Ras cells depleted of SCD2. Silencing of SCD2 also resulted in a significant downregulation of phosphorylated/activated ERK proteins, suggesting a positive regulation of the latter proteins by SCD2 in AKT/Ras cells. Similar to that observed in AKT/Ras cells depleted of SCD1, inactivation of SCD2 had no effect on phosphorylated/activated AKT levels.

**Figure 6:**
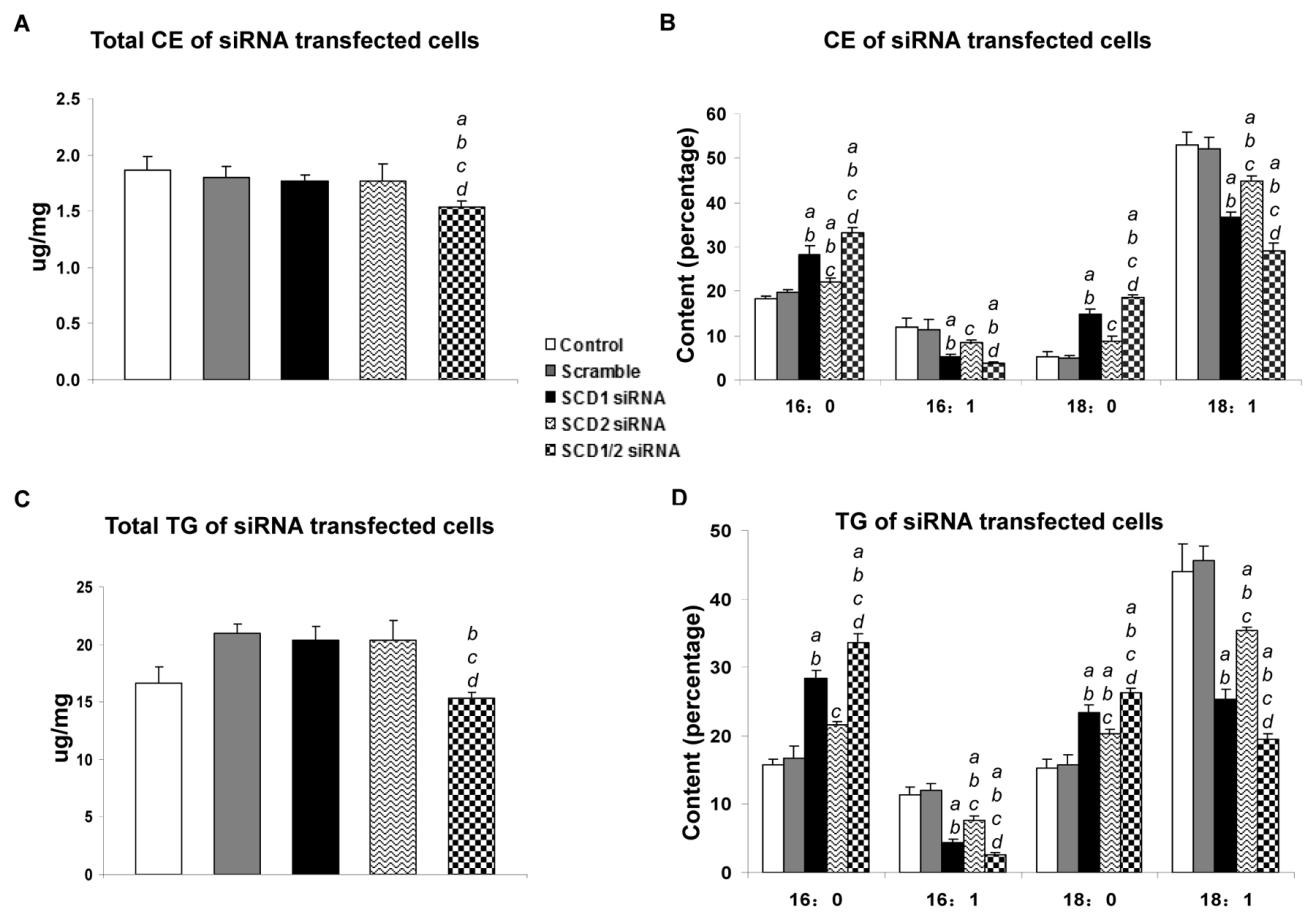
Lipid profiles of AKT/Ras cells transfected with scramble siRNA, SCD1 siRNA, SCD2 siRNA or SCD1 plus SCD2 siRNA. (A) Total Cholesterol ester (CE), (B) major fatty acids composition of Cholesterol ester, (C) Total Triglycerides (TG), and (D) major fatty acids composition of Triglycerides. Tukey-Kramer test: P < 0.005 *a*, vs. control (untreated cells); *b*, vs. scramble siRNA; *c*, vs. SCD1 siRNA; *d*, vs. SCD2 siRNA. Results 24 hours after silencing are shown. Experiments *in* *vitro* were conducted at least three times in triplicate.

Of note, concomitant silencing of SCD1 and SCD2 genes triggered a striking decline in cell proliferation and massive apoptosis in AKT/Ras cells (Figure 5A). At the molecular level, simultaneous inhibition of SCD1 and SCD2 led to a marked decrease in the levels of phosphorylated/activated AKT (Figure 5C). As concerns cellular lipid content, silencing of SCD1 resulted in a decrease of MUFA and increase of SFA percentage in the AKT/Ras cell line, in accordance with the *in vivo* data (Figure 6B and D). A similar, although less pronounced, decline of MUFA and rise of SFA was detected in AKT/Ras cells following SCD2 silencing (Figure 6B and D). Simultaneous inhibition of SCD1 and SCD2 genes led to a slightly further decrease of TG and CE levels as well as a slight, additional decrease in MUFA percentage when compared with silencing of either SCD1 or SCD2 alone (Figure 6B and D).

Altogether, the present data suggest the existence of compensatory loops in the SCD1/SCD2 axis in mouse HCC. Thus, suppression of both SCD1 and SCD2 genes might presumably be required to significantly impair AKT/Ras driven growth.

## Discussion

Most normal human tissues use dietary (exogenous) lipid for synthesis of new structural lipids, while *de novo* fatty acid synthesis is generally suppressed. In striking contrast, tumor cells frequently are addicted to *de novo* fatty acid synthesis [8]. Thus, the *de novo* lipogenesis pathway has become an attractive therapeutic target for cancer treatment [8]. FASN, the enzyme that catalyzes the *de novo* synthesis of long-chain fatty acids from acetyl-CoA and malonyl-CoA, has been the primary drug development focus of targeting *de novo* lipogenesis pathway [8]. However, FASN inhibitors have been shown to induce severe anorexia and weight loss, significantly limiting their clinical application potential [29].

Several studies demonstrate that stearoyl-CoA desaturases are the rate-determining enzymes in synthesis of MUFAs. As a consequence, it has been envisaged that SCD family proteins could be another possible target for inhibition of *de novo* lipogenesis based cancer treatment [29]. In accordance with this hypothesis, mounting evidence shows that inhibition of SCD1 leads to reduced cell proliferation and increased cell apoptosis in human cancer cell lines [30]. In most cases, the growth inhibitory activity against SCD1 could be rescued by adding to the cell culture medium MUFA, such as oleate, suggesting that the inhibition is specific for stearoyl-CoA desaturase.

Aberrant activation of PI3K/AKT/mTOR signaling cascade has been implicated in tumor development. It has been suggested that one of the major mechanisms of AKT/mTOR in promoting tumorigenesis is by regulating cancer metabolism, especially lipogenesis [31]. In particular, activated mTOR promotes SREBP-1 mRNA transcription as well as SREBP-1 processing, which in turn induces the expression of lipogenic pathway genes, including ACLY, ACC, FASN, and SCD1. Indeed, we have others have shown that activated AKT/mTOR induces SCD1 expression in cancers [19,32]. Conversely, SCD1 has been found to be required for AKT/mTOR induced tumor cell proliferation in human cancer cells. For example, it is reported that molecular or pharmacological inhibition of SCD1 led to the decreased AKT activation and growth inhibition in prostate cancer cells [33]. Similar results were observed in human HCC cell lines [19], suggesting a positive regulatory network between SCD1 expression and AKT/mTOR activation in cancer cells.

However, virtually all these experiments were performed *in vitro* using existing tumor cell lines. The requirement for SCD in tumor initiation and progression as well as the role of SCD in mediation AKT/mTOR induced tumor cell proliferation have not been tested using genetic models, such as *SCD1* null mice, to date. Here, we show that AKT induced hepatic steatosis and AKT/Ras driven hepatocarcinogenesis are not significantly affected by genetic ablation of *SCD1* in mice, demonstrating that mouse SCD1 *per se* is not essential for these biological processes. Furthermore, we found that SCD2, but not SCD3 and SCD4, is strongly upregulated in AKT/Ras liver tumor samples depleted of SCD1. Interestingly, SCD2 expression could not completely rescue stearoyl-CoA desaturase activity in the *SCD1* null tumor cells, as these tumors demonstrated significantly decreased MUFA content in their TG and CE lipid pools. However, this aberrant MUFA clearly did not affect AKT/Ras induced tumor development. On the other hand, using a primary HCC cell line isolated from an AKT/Ras tumor, we showed that silencing of SCD1 triggers the (presumably) compensatory upregulation of SCD2, which might confer survival advantages to SCD1-deprived cells. In support of this hypothesis, the concomitant suppression of SCD1 and SCD2 genes was able to efficiently restrain AKT/Ras cell growth *in vitro*. At the molecular level, our preliminary results suggest an oncogenic role for the ERK/MAPK pathway in AKT/Ras tumors from *SCD1* null mice. The specific contribution of the ERK/MAPK pathway in AKT/Ras hepatocarcinogenesis following SCD1 suppression as well as the mechanisms whereby SCD2 contributes to the activation of the ERK/MAPK cascade are currently under investigation.

Our findings have important implications for future studies. Genetic studies using mouse models are critical for validating cancer therapeutic targets. In the case of stearoyl-CoA desaturase, multiple SCD isoforms exist in the mice, and it is necessary to assay the expression of these SCD isoforms in different mouse tumor models. It may be necessary to delete multiple SCD isoforms in order to achieve efficient inhibition of stearoyl-CoA desaturase in tumors to observe tumor growth repression phenotypes. Indeed, based on the results obtained, the ablation of both *SCD1* and *SCD2* would be necessary in the liver tissue to ascertain the requirement of SCD proteins in hepatic steatosis and hepatocarcinogenesis. SCD2 has been successfully knocked out in mice [33]. However, both *SCD2* single knockout and *SCD1/SCD2* double knockout mice die soon after birth and, thus, are not suitable to study the requirement of SCD2 in liver tumor development. An alternative approach would be the generation of liver-specific, conditional *SCD1/SCD2* null mice, which could be then subjected to AKT or AKT/Ras hydrodynamic transfection. Another attracting alternative would be to apply short-hairpin (sh) RNA mediated silencing of SCD2 in combination with hydrodynamic injection in the liver of *SCD1* null mice. This technique has been successfully applied by Wuestefeld et al. to characterize genes which regulate liver regeneration [34], but its effectiveness on the study of liver tumor development *in vivo* remains to be addressed*.*

Another implication of the present study is that it might be important to completely inhibit SCD activity in human cancer cells in order to achieve sufficient tumor growth inhibition *in vivo*, whereas a partial SCD inhibitor might have limited therapeutic efficacy. Screening for small molecules which completely inhibit human SCD activity would be critical for successful therapeutic action against SCD in cancer treatment.

## References

[1] El-Serag HB, Rudolph KL (2007) Hepatocellular carcinoma: epidemiology and molecular carcinogenesis. Gastroenterology 132: 2557-2576. doi:10.1053/j.gastro.2007.04.061. PubMed: 17570226. 17570226 10.1053/j.gastro.2007.04.061

[2] Bruix J, Boix L, Sala M, Llovet JM (2004) Focus on hepatocellular carcinoma. Cancer Cell 5: 215-219. doi:10.1016/S1535-6108(04)00058-3. PubMed: 15050913. 15050913 10.1016/s1535-6108(04)00058-3

[3] Llovet JM, Bruix J (2008) Novel advancements in the management of hepatocellular carcinoma in 2008. J Hepatol 48 Suppl 1: S20-S37. doi:10.1016/S0168-8278(08)60049-5. PubMed: 18304676. 18304676 10.1016/j.jhep.2008.01.022

[4] Spangenberg HC, Thimme R, Blum HE (2009) Targeted therapy for hepatocellular carcinoma. Nat. Rev Gastroenterol Hepatol 6: 423-432. doi:10.1038/nrgastro.2009.86. 10.1038/nrgastro.2009.86 19488072

[5] Furuta E, Okuda H, Kobayashi A, Watabe K (2010) Metabolic genes in cancer: their roles in tumor progression and clinical implications. Biochim Biophys Acta 1805: 141-152. PubMed: 20122995. 20122995 10.1016/j.bbcan.2010.01.005 PMC2850259

[6] Cairns RA, Harris IS, Mak TW (2011) Regulation of cancer cell metabolism. Nat Rev Cancer 11: 85-95. doi:10.1038/nrc2981. PubMed: 21258394. 21258394 10.1038/nrc2981

[7] Mashima T, Seimiya H, Tsuruo T (2009) De novo fatty-acid synthesis and related pathways as molecular targets for cancer therapy. Br J Cancer 100: 1369-1372. doi:10.1038/sj.bjc.6605007. PubMed: 19352381. 19352381 10.1038/sj.bjc.6605007 PMC2694429

[8] Menendez JA, Lupu R (2007) Fatty acid synthase and the lipogenic phenotype in cancer pathogenesis. Nat Rev Cancer 7: 763-777. doi:10.1038/nrc2222. PubMed: 17882277. 17882277 10.1038/nrc2222

[9] Ntambi JM, Miyazaki M (2003) Recent insights into stearoyl-CoA desaturase-1. Curr Opin Lipidol 14: 255-261. doi:10.1097/00041433-200306000-00005. PubMed: 12840656. 12840656 10.1097/00041433-200306000-00005

[10] Hodson L, Fielding BA (2013) Stearoyl-CoA desaturase: rogue or innocent bystander? Prog Lipid Res 52: 15-42. doi:10.1016/j.plipres.2012.08.002. PubMed: 23000367. 23000367 10.1016/j.plipres.2012.08.002

[11] Igal RA (2010) Stearoyl-CoA desaturase-1: a novel key player in the mechanisms of cell proliferation, programmed cell death and transformation to cancer. Carcinogenesis 31: 1509-1515. doi:10.1093/carcin/bgq131. PubMed: 20595235. 20595235 10.1093/carcin/bgq131

[12] Hess D, Chisholm JW, Igal RA (2010) Inhibition of stearoylCoA desaturase activity blocks cell cycle progression and induces programmed cell death in lung cancer cells. PLOS ONE 5: e11394. doi:10.1371/journal.pone.0011394. PubMed: 20613975. 20613975 10.1371/journal.pone.0011394 PMC2894866

[13] Minville-Walz M, Pierre AS, Pichon L, Bellenger S, Fèvre C et al. (2010) Inhibition of stearoyl-CoA desaturase 1 expression induces CHOP-dependent cell death in human cancer cells. PLOS ONE 5: e14363. doi:10.1371/journal.pone.0014363. PubMed: 21179554. 21179554 10.1371/journal.pone.0014363 PMC3002938

[14] Morgan-Lappe SE, Tucker LA, Huang X, Zhang Q, Sarthy AV et al. (2007) Identification of Ras-related nuclear protein, targeting protein for Xenopus kinesin-like protein 2, and stearoyl-CoA desaturase 1 as promising cancer targets from an RNAi-based screen. Cancer Res 67: 4390-4398. doi:10.1158/0008-5472.CAN-06-4132. PubMed: 17483353. 17483353 10.1158/0008-5472.CAN-06-4132

[15] Hirsch HA, Iliopoulos D, Joshi A, Zhang Y, Jaeger SA et al. (2010) A transcriptional signature and common gene networks link cancer with lipid metabolism and diverse human diseases. Cancer Cell 17: 348-361. doi:10.1016/j.ccr.2010.01.022. PubMed: 20385360. 20385360 10.1016/j.ccr.2010.01.022 PMC2854678

[16] Düvel K, Yecies JL, Menon S, Raman P, Lipovsky AI et al. (2010) Activation of a metabolic gene regulatory network downstream of mTOR complex 1. Mol Cell 39: 171-183. doi:10.1016/j.molcel.2010.06.022. PubMed: 20670887. 20670887 10.1016/j.molcel.2010.06.022 PMC2946786

[17] Moon YA, Liang G, Xie X, Frank-Kamenetsky M, Fitzgerald K et al. (2012) The Scap/SREBP pathway is essential for developing diabetic fatty liver and carbohydrate-induced hypertriglyceridemia in animals. Cell Metab 15: 240-246. doi:10.1016/j.cmet.2011.12.017. PubMed: 22326225. 22326225 10.1016/j.cmet.2011.12.017 PMC3662050

[18] Porstmann T, Santos CR, Griffiths B, Cully M, Wu M et al. (2008) SREBP activity is regulated by mTORC1 and contributes to Akt-dependent cell growth. Cell Metab 8: 224-236. doi:10.1016/j.cmet.2008.07.007. PubMed: 18762023. 18762023 10.1016/j.cmet.2008.07.007 PMC2593919

[19] Calvisi DF, Wang C, Ho C, Ladu S, Lee SA et al. (2011) Increased lipogenesis, induced by AKT-mTORC1-RPS6 signaling, promotes development of human hepatocellular carcinoma. Gastroenterology 140: 1071-1083. doi:10.1053/j.gastro.2010.12.006. PubMed: 21147110. 21147110 10.1053/j.gastro.2010.12.006 PMC3057329

[20] Ho C, Wang C, Mattu S, Destefanis G, Ladu S et al. (2012) AKT (v-akt murine thymoma viral oncogene homologue 1) and N-Ras (neuroblastoma ras viral oncogene homolog) coactivation in the mouse liver promotes rapid carcinogenesis by way of mTOR (mammalian target of rapamycin complex 1), FOXM1 (forkhead box M1)/SKP2, and c-Myc pathways. Hepatology 55: 833-845. doi:10.1002/hep.24736. PubMed: 21993994. 21993994 10.1002/hep.24736 PMC3269553

[21] Fan L, Xu C, Wang C, Tao J, Ho C et al. (2012) Bmi1 is required for hepatic progenitor cell expansion and liver tumor development. PLOS ONE 7: e46472. doi:10.1371/journal.pone.0046472. PubMed: 23029524. 23029524 10.1371/journal.pone.0046472 PMC3460872

[22] Dobrzyn P, Dobrzyn A, Miyazaki M, Cohen P, Asilmaz E et al. (2004) Stearoyl-CoA desaturase 1 deficiency increases fatty acid oxidation by activating AMP-activated protein kinase in liver. Proc Natl Acad Sci U S A 101: 6409-6414. doi:10.1073/pnas.0401627101. PubMed: 15096593. 15096593 10.1073/pnas.0401627101 PMC404058

[23] Lee SA, Ho C, Roy R, Kosinski C, Patil MA et al. (2008) Integration of genomic analysis and in vivo transfection to identify sprouty 2 as a candidate tumor suppressor in liver cancer. Hepatology 47: 1200-1210. PubMed: 18214995. 18214995 10.1002/hep.22169

[24] Carlson CM, Frandsen JL, Kirchhof N, McIvor RS, Largaespada DA (2005) Somatic integration of an oncogene-harboring Sleeping Beauty transposon models liver tumor development in the mouse. Proc Natl Acad Sci U S A 102: 17059-17064. doi:10.1073/pnas.0502974102. PubMed: 16286660. 16286660 10.1073/pnas.0502974102 PMC1287966

[25] Folch J, Lees M, Sloane Stanley GH (1957) A simple method for the isolation and purification of total lipides from animal tissues. J Biol Chem 226: 497-509. PubMed: 13428781. 13428781

[26] Morrison WR, Smith LM (1964) Preparation of Fatty Acid Methyl Esters and Dimethylacetals from Lipids with Boron Fluoride--Methanol. J Lipid Res 5: 600-608. PubMed: 14221106. 14221106

[27] Ntambi JM, Miyazaki M, Dobrzyn A (2004) Regulation of stearoyl-CoA desaturase expression. Lipids 39: 1061-1065. doi:10.1007/s11745-004-1331-2. PubMed: 15726820. 15726820 10.1007/s11745-004-1331-2

[28] Chu K, Miyazaki M, Man WC, Ntambi JM (2006) Stearoyl-coenzyme A desaturase 1 deficiency protects against hypertriglyceridemia and increases plasma high-density lipoprotein cholesterol induced by liver X receptor activation. Mol Cell Biol 26: 6786-6798. doi:10.1128/MCB.00077-06. PubMed: 16943421. 16943421 10.1128/MCB.00077-06 PMC1592860

[29] López M, Lelliott CJ, Tovar S, Kimber W, Gallego R et al. (2006) Tamoxifen-induced anorexia is associated with fatty acid synthase inhibition in the ventromedial nucleus of the hypothalamus and accumulation of malonyl-CoA. Diabetes 55: 1327-1336. doi:10.2337/db05-1356. PubMed: 16644689. 16644689 10.2337/db05-1356

[30] Mason P, Liang B, Li L, Fremgen T, Murphy E et al. (2012) SCD1 inhibition causes cancer cell death by depleting mono-unsaturated fatty acids. PLOS ONE 7: e33823. doi:10.1371/journal.pone.0033823. PubMed: 22457791. 22457791 10.1371/journal.pone.0033823 PMC3310881

[31] Bakan I, Laplante M (2012) Connecting mTORC1 signaling to SREBP-1 activation. Curr Opin Lipidol 23: 226-234. doi:10.1097/MOL.0b013e328352dd03. PubMed: 22449814. 22449814 10.1097/MOL.0b013e328352dd03

[32] Luyimbazi D, Akcakanat A, McAuliffe PF, Zhang L, Singh G et al. (2010) Rapamycin regulates stearoyl CoA desaturase 1 expression in breast cancer. Mol Cancer Ther 9: 2770-2784. doi:10.1158/1535-7163.MCT-09-0980. PubMed: 20876744. 20876744 10.1158/1535-7163.MCT-09-0980 PMC2965451

[33] Fritz V, Benfodda Z, Rodier G, Henriquet C, Iborra F et al. (2010) Abrogation of de novo lipogenesis by stearoyl-CoA desaturase 1 inhibition interferes with oncogenic signaling and blocks prostate cancer progression in mice. Mol Cancer Ther 9: 1740-1754. doi:10.1158/1535-7163.MCT-09-1064. PubMed: 20530718. 20530718 10.1158/1535-7163.MCT-09-1064 PMC3315476

[34] Wuestefeld T, Pesic M, Rudalska R, Dauch D, Longerich T et al. (2013) A Direct in vivo RNAi screen identifies MKK4 as a key regulator of liver regeneration. Cell 153: 389-401. doi:10.1016/j.cell.2013.03.026. PubMed: 23582328. 23582328 10.1016/j.cell.2013.03.026

